# A Preliminary Study to Use SUVmax of FDG PET-CT as an Identifier of Lesion for Artificial Intelligence

**DOI:** 10.3389/fmed.2021.647562

**Published:** 2021-04-28

**Authors:** Kenji Hirata, Osamu Manabe, Keiichi Magota, Sho Furuya, Tohru Shiga, Kohsuke Kudo

**Affiliations:** ^1^Department of Diagnostic Imaging, Hokkaido University Graduate School of Medicine, Sapporo, Japan; ^2^Department of Nuclear Medicine, Hokkaido University Hospital, Sapporo, Japan; ^3^Department of Diagnostic and Interventional Radiology, Hokkaido University Hospital, Sapporo, Japan; ^4^Department of Radiology, Saitama Medical Center, Jichi Medical University, Saitama, Japan; ^5^Division of Medical Imaging and Technology, Hokkaido University Hospital, Sapporo, Japan; ^6^Global Center for Biomedical Science and Engineering, Faculty of Medicine, Hokkaido University, Sapporo, Japan

**Keywords:** maximum of standardized uptake value, SUVmax, identifier, FDG PET, diagnostic report, artificial intelligence

## Abstract

**Background:** Diagnostic reports contribute not only to the particular patient, but also to constructing massive training dataset in the era of artificial intelligence (AI). The maximum standardized uptake value (SUVmax) is often described in daily diagnostic reports of [^18^F] fluorodeoxyglucose (FDG) positron emission tomography (PET) – computed tomography (CT). If SUVmax can be used as an identifier of lesion, that would greatly help AI interpret diagnostic reports. We aimed to clarify whether the lesion can be localized using SUVmax strings.

**Methods:** The institutional review board approved this retrospective study. We investigated a total of 112 lesions from 30 FDG PET-CT images acquired with 3 different scanners. SUVmax was calculated from DICOM files based on the latest Quantitative Imaging Biomarkers Alliance (QIBA) publication. The voxels showing the given SUVmax were exhaustively searched in the whole-body images and counted. SUVmax was provided with 5 different degrees of precision: integer (e.g., 3), 1st decimal places (DP) (3.1), 2nd DP (3.14), 3rd DP (3.142), and 4th DP (3.1416). For instance, when SUVmax = 3.14 was given, the voxels with 3.135 ≤ SUVmax < 3.145 were extracted. We also evaluated whether local maximum restriction could improve the identifying performance, where only the voxels showing the highest intensity within some neighborhood were considered. We defined that “identical detection” was achieved when only single voxel satisfied the criterion.

**Results:** A total of 112 lesions from 30 FDG PET-CT images were investigated. SUVmax ranged from 1.3 to 49.1 (median = 5.6). Generally, when larger and more precise SUVmax values were given, fewer voxels satisfied the criterion. The local maximum restriction was very effective. When SUVmax was determined to 4 decimal places (e.g., 3.1416) and the local maximum restriction was applied, identical detection was achieved in 33.3% (lesions with SUVmax < 2), 79.5% (2 ≤ SUVmax < 5), and 97.8% (5 ≤ SUVmax) of lesions.

**Conclusion:** In this preliminary study, SUVmax of FDG PET-CT could be used as an identifier to localize the lesion if precise SUVmax is provided and local maximum restriction was applied, although the lesions showing SUVmax < 2 were difficult to identify. The proposed method may have potential to make use of diagnostic reports retrospectively for constructing training datasets for AI.

## Introduction

The clinical usefulness of positron emission tomography (PET) using [^18^F]-fluorodeoxyglucose (FDG) has been well-established in oncology (1, 2). In addition to visual assessment (qualitative analysis), several quantitative measurements have been used to express the degree of FDG uptake. Among them, the standardized uptake value (SUV) has long been used as the de facto standard. To our knowledge, SUV was first extensively used around 1991 (3). In the initial years of its use, SUV was also known as the differential uptake ratio (4) or dose uptake ratio (5). The in-lesion maximum of SUV, or SUVmax, has frequently been used since 1999. By 2009 SUVmax had become the most frequently used measurement by far, with 6-fold more frequent use compared to the next most-often used measurement, according to a comprehensive review (6). Although SUV is most commonly calculated as the radioactivity concentration normalized to injection dosage and body weight, other definitions include the radioactivity concentration normalized to the body surface area (7), to lean body mass (6), and to blood glucose (8). In addition to SUVmax, metabolic tumor volume and total lesion glycolysis have been extensively investigated in recent studies and the evidence has been increasingly accumulated (9, 10). These volumetric measurements are, however, affected by the method of tumor boundary delineation, degrading inter-operator reproducibility. In contrast, SUVmax has theoretically highest inter-operator reproducibility. Many lines of evidence have demonstrated the usefulness of SUVmax for differential diagnosis, treatment response prediction, and prognosis (11).

A total of 0.6 million FDG PET studies were performed in Japan in 2017 (12), and we think that diagnostic reports were written for most of them. Describing intensity of FDG uptake either using SUVmax or qualitatively has been recommended (13). Diagnostic reports not only contribute greatly to helping the attending physician interpret the image and diagnose the disease, but also prevent important findings from being overlooked. More recently, in the era of artificial intelligence (AI), the importance of training data is increasing. Collectively, diagnostic reports form a highly useful and efficient training database (14–18).

**Figure 1:**
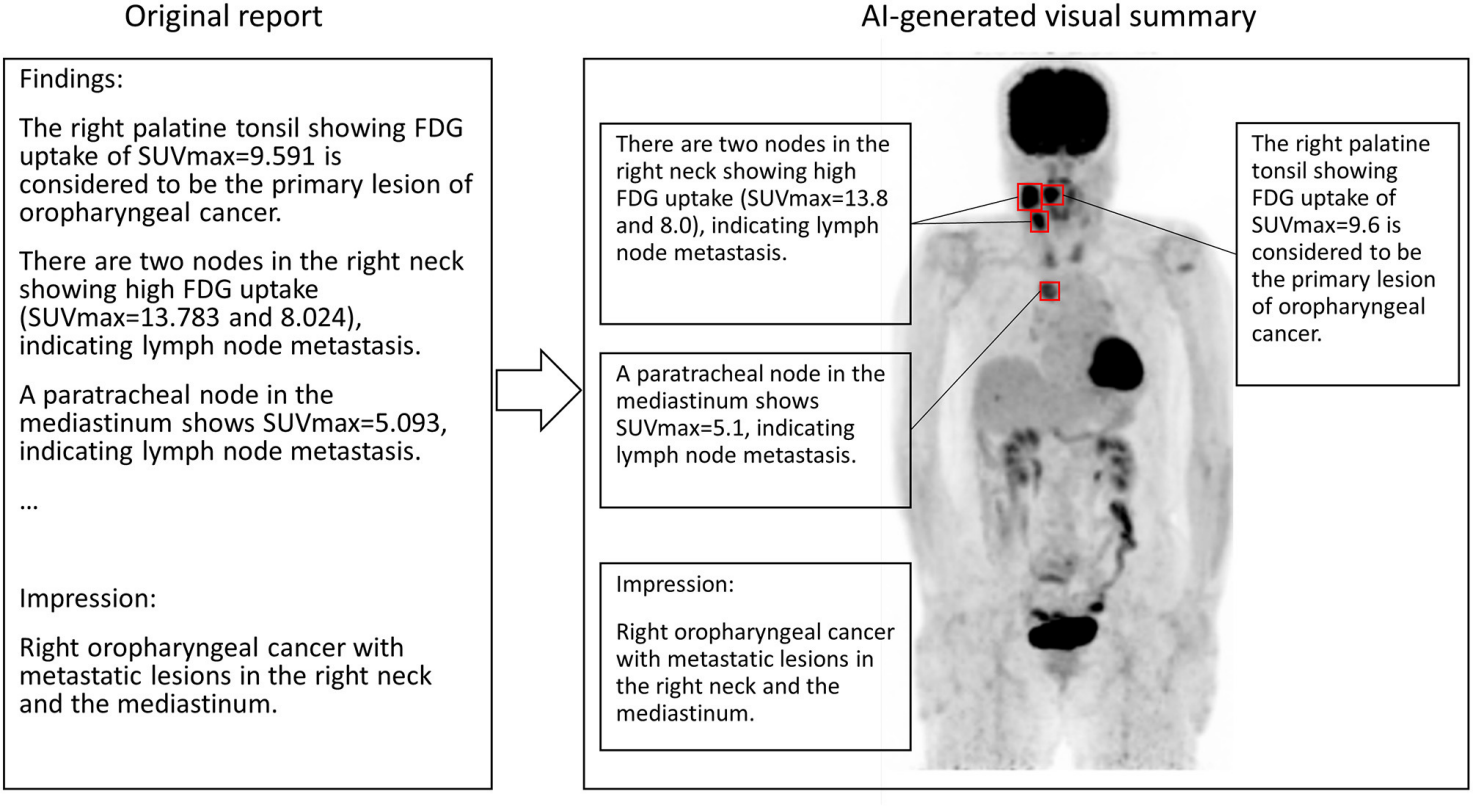
A conceptual image of AI generating the visual summary of the report of FDG PET. SUVmax in the sentence appearing in the report text is used for localization. In this case, the primary lesion (right palatine tonsil) and metastatic nodes show high FDG uptakes. Note that SUVmax should be round before attending physicians read the report.

We hypothesized that if the SUVmax described in diagnostic reports was sufficiently precise, it might contribute to localization of the lesion, because there should be a limited number of voxels showing the same SUVmax in the entire image. In other words, we thought that SUVmax could be used as an identifier of the lesion. Thus, in this study, we aimed to clarify whether it would be possible to identify the lesion location using the SUVmax under various conditions by varying the degree of SUVmax precision and applying local maximum restriction. Such a technique could also be used to realize an automated system to generate a visual summary of the diagnostic report (Figure 1).

## Materials and Methods

### Study Subjects

This retrospective observation study was approved by the institutional review board (approval no. 017-0454). The requirement of written informed consent from each patient was waived because of the study's retrospective nature. We confirmed that all methods were carried out in line with the relevant guidelines and regulations. A total of 30 PET-CT scans (sequential examinations for each scanner) were investigated in this study. No more than one scan was included for each patient. All the images were acquired between April 2019 and November 2019. Images were evaluated visually, and included to the study population if there were any pathological FDG uptakes in visual analysis until the number of scans reached 10 for each scanner. When all the FDG accumulation masses were considered physiological, the case was excluded. Note that not only uptake due to pathological malignancy but also malignancy-suspected and inflammatory uptakes were included in the analysis. In cases more than 5 uptakes were found, a maximum of 5 uptakes that showed highest values were recorded for a patient, based on RECIST 1.1 (19). Two experienced nuclear medicine physicians visually evaluated all the images. In case the interpretations of the two physicians differed, the final interpretation was reached by discussion.

### PET-CT Image Acquisition and Reconstruction

In this study, we investigated images acquired with 3 different PET-CT scanners made by 2 different manufacturers.

Scanner 1 was a Biograph 64 True Point PET-CT (Siemens, Munich, Germany). The transaxial and axial fields of view were 68.4 and 21.6 cm, respectively. Emission data was acquired for 180 s per bed. Images were reconstructed using the OSEM algorithm with point spread function correction. Time-of-flight of photons was not measurable with the scanner. The reconstructed images had a matrix size of 168 × 168 and a voxel size of 4.1 × 4.1 × 2.0 mm.

Scanner 2 was a GEMINI TF64 PET-CT (Philips, Amsterdam, Netherlands). The transaxial and axial fields of view were 57.6 and 18.0 cm, respectively. Emission data was acquired for 60–180 s per bed depending on patient weight and injected dosage. Images were reconstructed using the OSEM algorithm reinforced with the time-of-flight algorithm. Point spread function correction was not applied. The reconstructed images had a matrix size of 144 × 144 and a voxel size of 4.0 × 4.0 × 4.0 mm.

Scanner 3 was a Vereos PET-CT (Philips, Amsterdam, Netherlands), which was the newest scanner of the three and equipped with digital photon counting detectors (20). The transaxial and axial fields of view were 67.6 and 16.4 cm, respectively (20). Emission data was acquired for 120–180 s per bed depending on patient weight and injected dosage. Images were reconstructed using the OSEM algorithm. Both the time-of-flight algorithm and point spread function correction were applied. The reconstructed images had a matrix size of 256 × 256 and a voxel size of 2.0 × 2.0 × 2.0 mm.

The number of voxels in the z-direction (i.e., cranio-caudal direction) ranged from 234 to 553, resulting in the final number of voxels ranging from 4.85 × 10^6^ to 4.41 × 10^7^. CT images were used for attenuation correction for all the scanners and for visual assessment, but were not analyzed quantitatively in the current study. All patients fasted for ≥6 h before the injection of FDG (~4 MBq/kg), and the emission scanning was initiated basically around 60 min post-injection. One scan was acquired 95 min post-injection due to mechanical troubles. Fasting blood sugar was confirmed to be smaller than 200 mg/dl in each study.

### SUVmax Calculation

Commercially available DICOM viewers/PET viewers do not display SUVmax to 4 decimal places (DP) or higher. In order to obtain the ground truth of SUVmax, we modified Metavol software package, which we previously developed for PET-CT volumetric analysis (21). We used Windows 10, Microsoft Visual Studio Community 2019 Version 16.4.0, C# 8.0 language, .NET Core 3.1, and fo-dicom 4.0.3 for modifying Metavol. For instance, in the case that the true SUVmax is 3.14159, the modified Metavol will display it as it is, whereas XTREK VIEW software (J-MAC SYSTEM, Sapporo, Japan) will display it as 3.142. A nuclear medicine physician measured SUVmax by placing a spherical volume of interest (VOI) whose diameter can be changed by the operator. Another nuclear medicine physician independently confirmed all the values of SUVmax.

After the VOI definition, SUVmax was calculated based on the newest QIBA publication (22). Briefly, in Biograph64 and Vereos, the radioactivity concentration c (Bq/ml) was calculated as follows:

$$\begin{array}{l} c=\rho \cdot s+i. \end{array}$$

Here, ρ represents the raw pixel value that was stored with DICOM tag of (7FE0,0010) with each voxel expressed in a 16-bit integer. s represents the *rescale slope*, which is stored as a float value at (0028, 1053). i represents the *rescale intercept*, which is stored as a float value at (0028, 1052). Next, decay-corrected injection dosage D_c_ was calculated as follows:

$$\begin{array}{l} D_{\text{c}}=D_{0}\cdot {\left(1/2\right)}^{\left(\text{Ta}-Ti\right)/h}. \end{array}$$

Here, D_0_ represents the *radionuclide total dose* (i.e., injected dosage of FDG) (Bq) stored as a float value at (0018, 1074). T_a_ represents *acquisition time* stored at (0008, 0032). T_i_ represents the *radiopharmaceutical start time* (i.e., injection time) stored at (0018, 1072). Both times are stored in a “hhmmss” form string, and thus conversion to second is needed. h represents the *radionuclide half-life* (second) stored as a float value at (0018, 1075).

Finally, SUV was calculated as follows:

$$\begin{array}{l} SUV=c\cdot w/D_{\text{c}}. \end{array}$$

Here, w represents the *patient's weight* (g), which is stored in kilograms at (0010, 1030) and thus must be multiplied by 1000.

The SUV calculation was much simpler in GEMINI TF64, as follows:

$$\begin{array}{l} SUV=\left(\rho \cdot s+i\right)\cdot p. \end{array}$$

Here, p represents the *Philips Factor* (float) stored as a float value at (7053, 1000). The value of i was 0 for all the GEMINI TF64 examinations investigated in the current study.

### Lesion Localization

**Figure 2:**
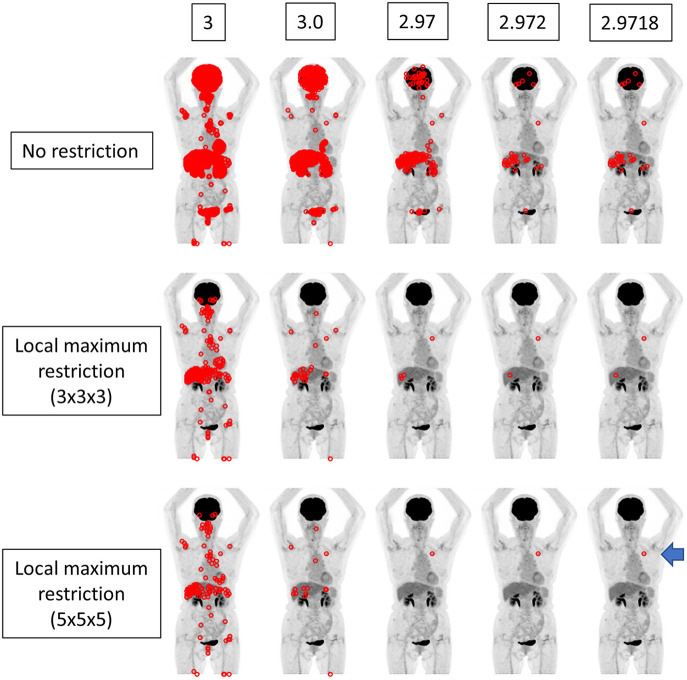
The findings for a patient who underwent FDG PET-CT for lung nodules. The true SUVmax of the nodule in the left upper lobe was 2.97177 (arrow). When local maximum restriction was not applied, 21031, 2176, 210, 33, and 33 voxels were extracted for 3, 3.0, 2.97, 2.972, and 2.9718, respectively. When 3 × 3 × 3 local maximum restriction was applied, 254, 32, 4, 2, and 2 voxels were extracted. When 5 × 5 × 5 local maximum restriction was applied, 126, 14, 1, 1, and 1 voxel(s) were extracted, achieving identical detection.

**Figure 3:**
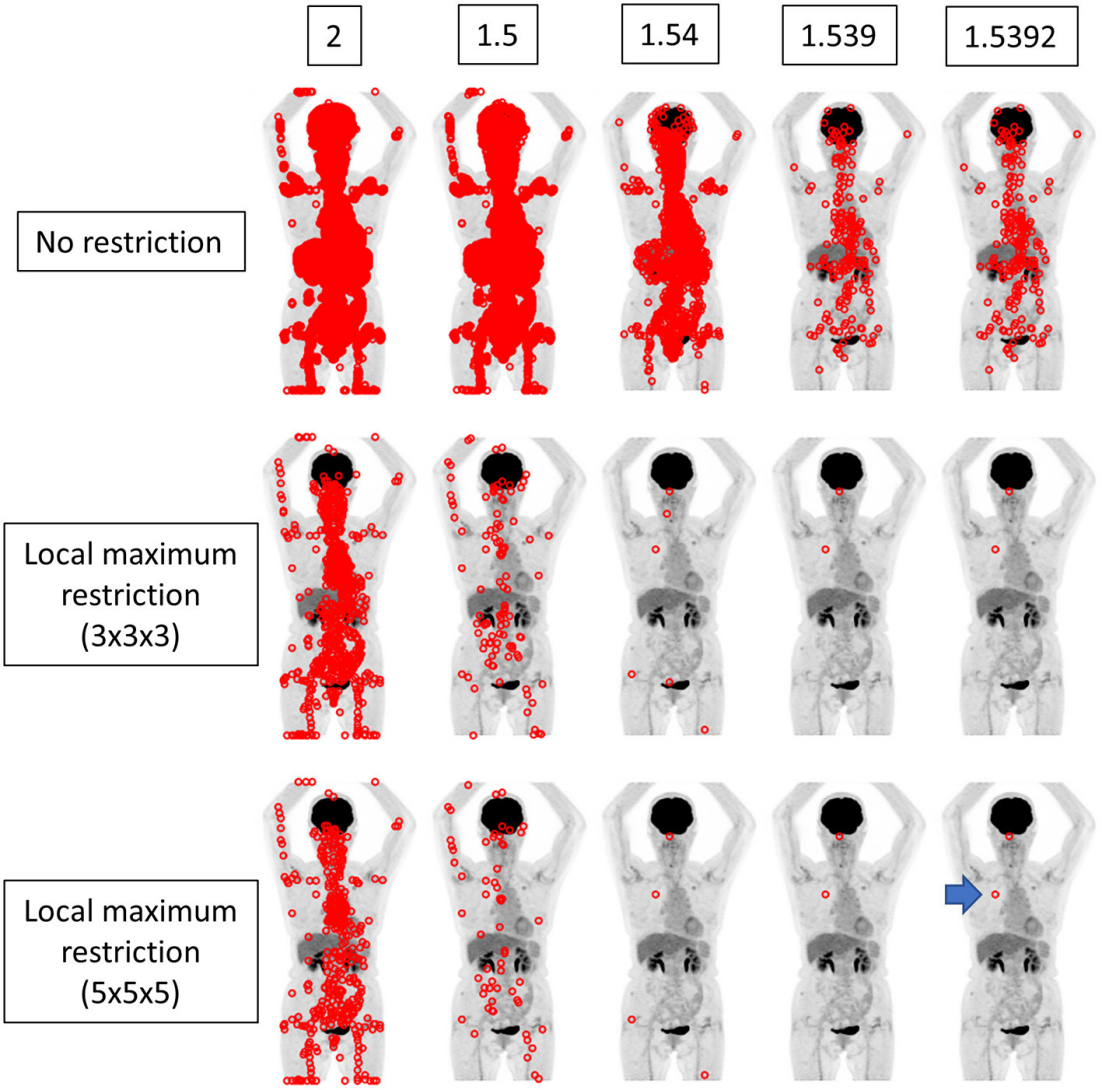
The same case as depicted in Figure 2. The true SUVmax of the nodule in the right upper lobe was 1.53924 (arrow). When local maximum restriction was not applied, 74952, 13442, 1427, 198, and 198 voxels were extracted for 2, 1.5, 1.54, 1.539, and 1.5392, respectively. When 3 × 3 × 3 local maximum restriction was applied, 782, 104, 6, 2, and 2 voxels were extracted. When 5 × 5 × 5 local maximum restriction was applied, 410, 60, 4, 2, and 2 voxels were extracted. Thus, identical detection was not achieved for this lesion.

**Figure 4:**
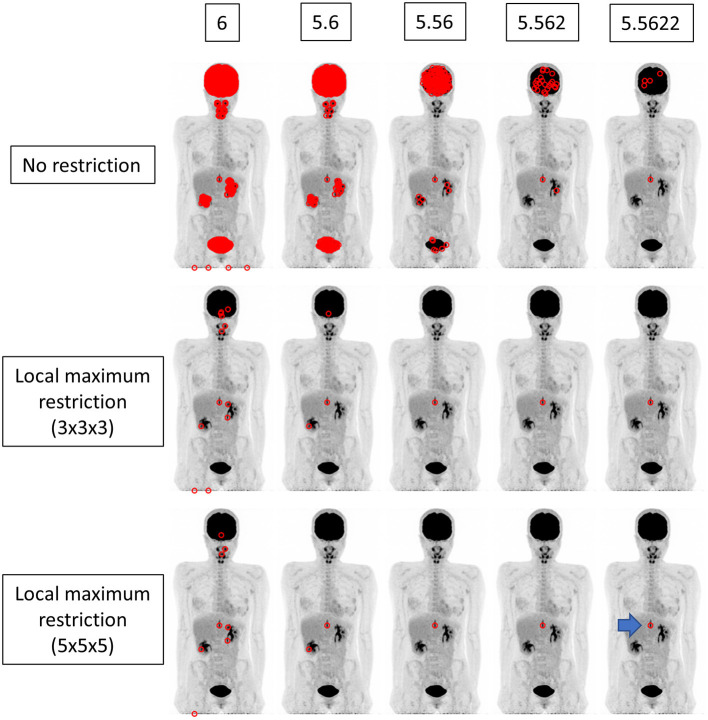
The findings for a patient who underwent FDG PET-CT for a spinal code lesion. The true SUVmax of the nodule in the spinal code lesion was 5.56218 (arrow). When local maximum restriction was not applied, 21116, 1953, 186, 25, and 5 voxels were extracted for 6, 5.6, 5.56, 5.562, and 5.5622, respectively. When 3 × 3 × 3 local maximum restriction was applied, 12, 3, 1, 1, and 1 voxel(s) were extracted. When 5 × 5 × 5 local maximum restriction was applied, 8, 2, 1, 1, and 1 voxel(s) were extracted, achieving identical detection.

We implemented a function that searches voxels satisfying the given SUV range and illustrate the locations in the whole body image (Figures 2–4). The SUV range was determined as follows. When “3” was provided by the operator, the range was considered to be 2.5 ≤ SUV < 3.5. When “3.1” was provided, the voxels satisfying 3.05 ≤ SUV < 3.15 were picked out, and so forth. Thus, the more precise the provided value of SUVmax (i.e., more digits) was, the narrower the range of SUV applied to extract voxels was. We compared the results from integer precision to 4th DP precision. Note that we do not show the results of 5th DP precision because there were no cases in which 5th DP precision improved the identification rate compared to 4th DP precision.

We performed experiments in different settings. First, the voxels within the range were extracted simply. Then, local maximum restriction was added to discard the voxel that was adjacent to the higher-value voxel, because such a voxel cannot have SUVmax. For local maximum restriction, milder restriction and stricter restriction were tested. Milder restriction was a condition under which the voxel must be highest in the 3 × 3 × 3 cube. Stricter restriction was a condition under which the voxel must be highest in the 5 × 5 × 5 cube.

Here, we defined that “identical detection” was achieved when only 1 voxel satisfied the criterion.

### Statistical Analysis

The relationship between SUVmax vs. the number of voxels detected (*N*) was estimated using Pearson's correlation coefficient of the log of SUVmax vs. the log of *N*. The effects of the precision of SUVmax, i.e., the number of digits after the decimal point, and local maximum restriction on the rate of identical detection were tested using a chi-square test. *P*-values < 0.05 were considered statistically significant.

## Results

Patient characteristics are summarized in Table 1. Diagnosis and lesion locations are summarized in Table 2. In this study population, head-and-neck cancer was the most common diagnosis, and the mediastinal and hilar lymph nodes were the most frequent locations. In the 112 lesions investigated, SUVmax ranged from 1.3 to 49.1, with median and interquartile range (IQR) values of 5.6 and 5.2, respectively. SUVmax was significantly higher for Vereos than for Biograph64 and TF64 (*P* < 0.01 and *P* < 0.05, respectively; Supplementary Figure 1), which could be due to variability of diseases and scanner characteristics. The numbers of lesions for Biograph64, GEMINI TF64, and Vereos were 37, 37, and 38, respectively.

**Table 1:** Patient characteristics.

	**Minimum**	**25-percentile**	**50-percentile (median)**	**75-percentile**	**Maximum**
Age (year)^*^	11	62.25	69	75	86
Body weight (kg)	35.6	50.75	54.5	65.7	78.5
Fasting blood sugar (mg/dl)^**^	82	92.25	100.5	107	182
Injected dosage (MBq/body)	140.1	226.6	242.4	287.4	348.0
Injected dosage (MBq/kg)	2.97	4.33	4.41	4.47	4.74
Fasting time (hour)	5.5	7.0	15.5	17.0	22.0
Uptake time (min)^***^	53	55	56	60.5	95

* *1 (3%) patient was younger than 20 years old*.

** *4 (13%) patients were diagnosed as having diabetes*.

*** *uTime duration between FDG injection and image acquisition start*.

**Table 2:** Diagnosis and lesion sites.

**Diagnosis**	**Number of patients**	**Site**	**Number of lesions**
Head and neck cancer	11	Mediastinal and hilar nodes	29
Lung cancer	5	Bone	20
Colorectal cancer	4	Neck and subclavian nodes	17
Malignant lymphoma	2	Lung	16
Primary unknown cancer	2	Abdominal nodes	6
Spinal cord tumor	2	Nasal cavity and pharynx	4
Myelitis	1	Intestine	4
Hepatobiliary cancer	1	Breast	3
Mediastinal tumor	1	Spinal cord	3
Sarcoidosis	1	Other soft tissues	3
		Axillary nodes	2
		Liver	1
		Inguinal nodes	1
		Adrenal gland	1
		Parotid gland	1
		Peripheral nerve	1
Total	30		112

**Figure 5:**
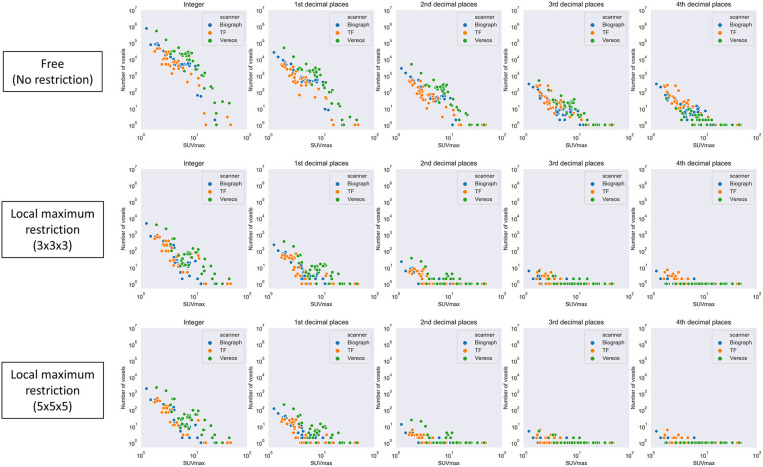
The number of voxels extracted by a given SUVmax with various levels of precision. Top row, local maximum restriction was not applied; middle row, 3 × 3 × 3 local maximum restriction was applied; bottom row, 5 × 5 × 5 local maximum restriction was applied.

First, local maximum restriction was not applied. A number of voxels were identified corresponding to the given SUVmax (Figure 5, top row). Generally, when a larger SUVmax was given, a smaller number of voxels was detected (0.83 < |*r*| < 0.84, *P* < 10^−28^). When the SUVmax was given with 10-fold greater precision, an ~0.1-fold number of voxels were extracted, as expected theoretically.

**Figure 6:**
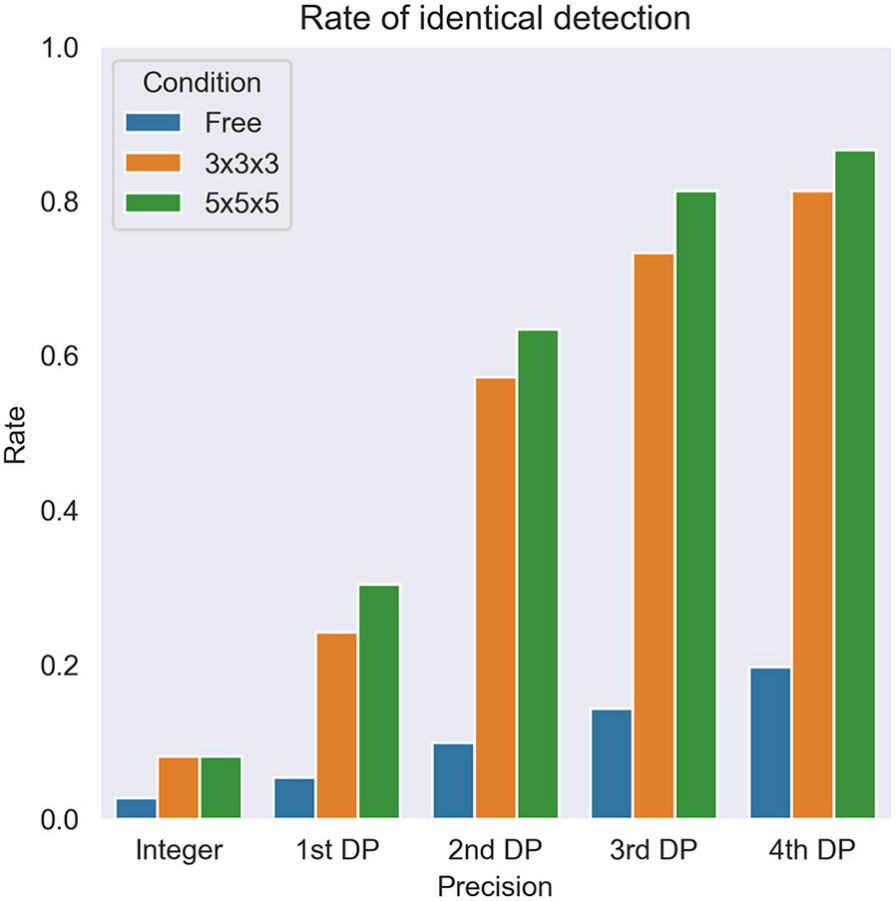
The overall rate of identical detection of the lesion. DP, decimal places. Free, 3 × 3 × 3, and 5 × 5 × 5 express no restriction and each local maximum restriction.

Next, local maximum restriction was applied. Both 3 × 3 × 3 and 5 × 5 × 5 local maximum restriction reduced the number of extracted voxels up to 1/1000 (Figure 5, middle and bottom rows). More specifically, the rate of identical detection increased when the given SUVmax was more precise and local maximum restriction was stricter (Figure 6). For instance, while identical detection was successful only in 2.7% of patients when integer precision and no restriction were used, the success rate was elevated to 86.6% when 4th DP precision and 5 × 5 × 5 local maximum restriction were used. The effects of 5 × 5 × 5 over 3 × 3 × 3 local maximum restriction were observed as shown in Figure 6, except for integer precision, although none of the differences between 5 × 5 × 5 vs. 3 × 3 × 3 local maximum restriction reached the level of statistical significance (*P* > 0.05).

**Figure 7:**
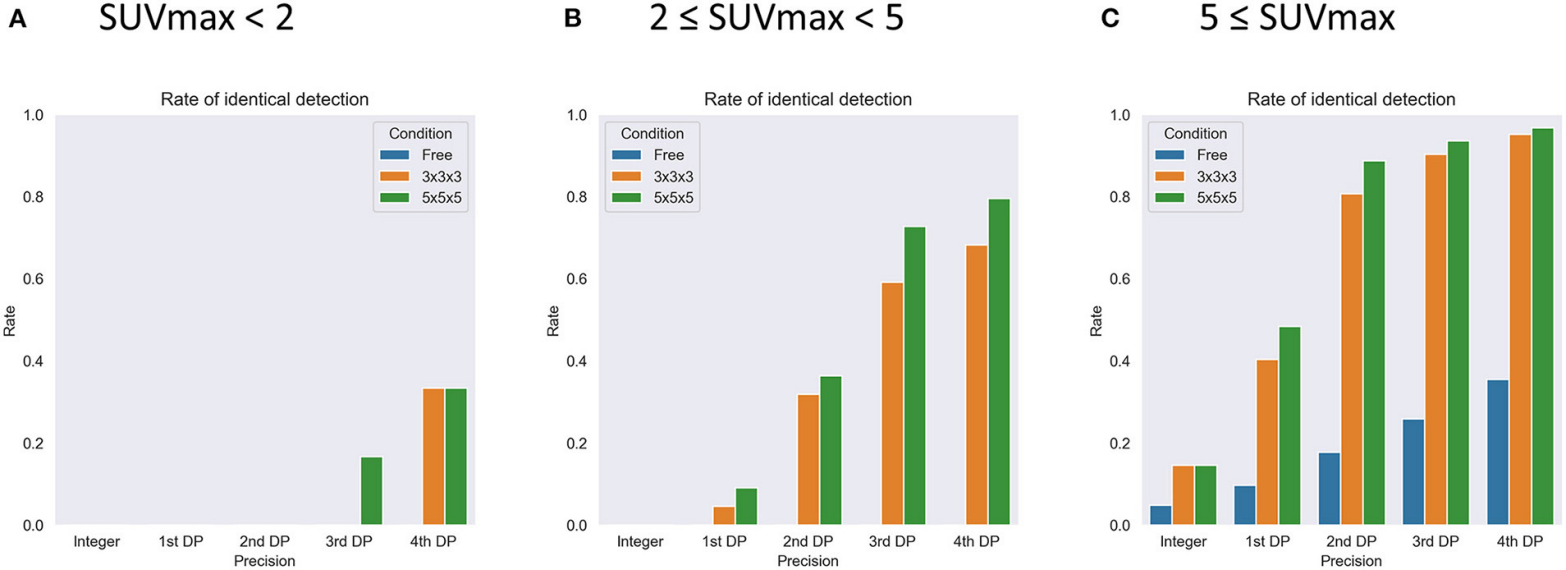
The results of sub-group analysis of the rate of identical detection of the lesions with SUVmax < 2 (**A**, *N* = 6), 2 < SUVmax < 5 (**B**, *N* = 44), and 5 < SUVmax (**C**, *N* = 62). DP, decimal places. Free, 3 × 3 × 3, and 5 × 5 × 5 express no restriction and each local maximum restriction.

**Figure 8:**
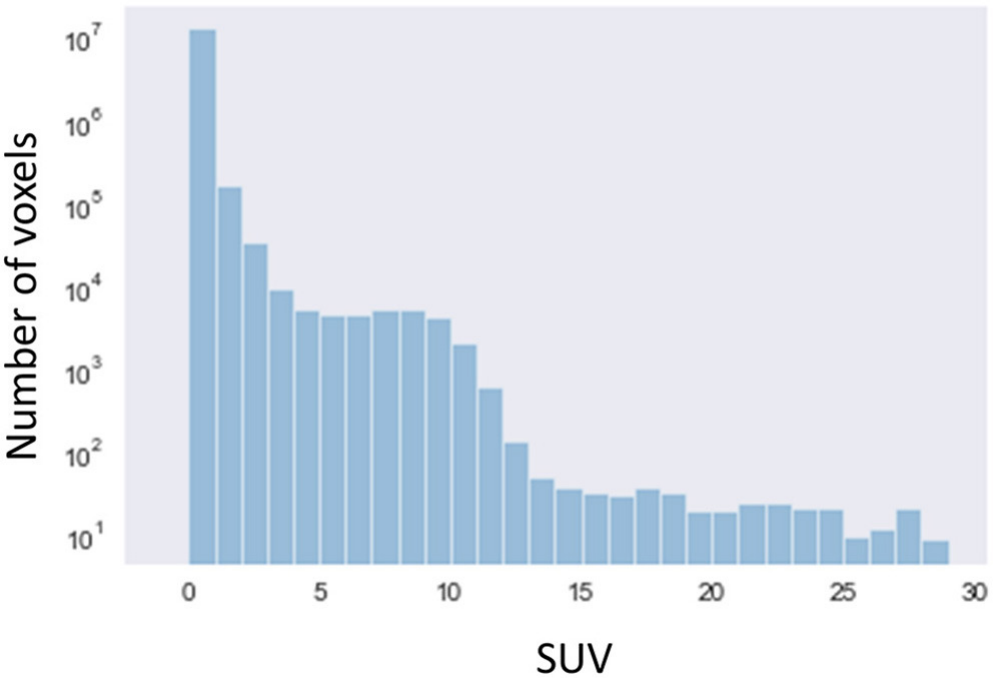
A histogram of SUV over the whole-body image of a patient (semi-log plot).

For sub-analysis, all lesions were categorized as low (SUVmax < 2, *N* = 6), medium (2 ≤ SUVmax < 5, *N* = 44), or high (5 ≤ SUVmax, *N* = 62) uptake lesions. The rate of identical detection was low (33.3%) for the low uptake group even under the best conditions, although the medium (79.5%) and high (96.8%) uptake groups achieved high rates (Figure 7). To investigate the underlying mechanisms for this difference, we drew a histogram of SUV over the whole-body image of a patient (Figure 8). In this case, the frequency exponentially decreased when SUVmax increased, as 98.13% of voxels showed 0 ≤ SUV < 1, 1.28% showed 1 ≤ SUV < 2, 0.37% showed 2 ≤ SUV < 5, and 0.21% showed 5 ≤ SUV.

In addition, Table 3 summarizes the statistical analysis to search variables affecting the rate of identical detection of the lesion. In this analysis, all the lesions were categorized into 2 groups using the median of the variable as the cut-off. As the results, young age (*p* = 0.01) and large injected dosage (MBq/kg, *p* = 0.005) were significant factors for high rate of identical detection of the lesion. Note that injected dosage per body (MBq/body) was not a significant factor. We found that there were no significant correlations between SUVmax and patient age (*r* = −0.01) or between SUVmax and injected dosage (MBq/kg, *r* = 0.17).

**Table 3:** Variables affecting the rate of identical detection of the lesion.

	** <Median**			**≥Median**			p^*^
	**Number of lesions (A)**	**Number of identical detection (B)**	**Rate (B/A)**	**Number of lesions (A)**	**Number of identical detection (B)**	**Rate (B/A)**	
Age (year)	59	56	94.9%	53	41	77.4%	0.01
Body weight (kg)	58	52	89.7%	54	45	83.3%	0.41
Fasting blood sugar (mg/dl)	53	48	90.6%	59	49	83.1%	0.28
Injected dosage (MBq/body)	54	49	90.7%	58	48	82.8%	0.27
Injected dosage (MBq/kg)	51	39	76.5%	61	58	95.1%	0.005
Fasting time (hour)	47	41	87.2%	65	56	86.2%	1.0
Uptake time (min)^**^	36	30	83.3%	76	67	88.2%	0.56

* *p-values were calculated Fisher's exact test*.

** *Time duration between FDG injection and image acquisition start*.

## Discussion

In this retrospective study, we aimed to clarify whether SUVmax can be used as a lesion identifier to localize the voxel in the whole-body image of FDG PET. We observed that SUVmax successfully localized the voxel for >80% examinations in the case that SUVmax was given to the 3rd or higher DP and local maximum restriction (5 × 5 × 5) was applied. However, the sub-analysis showed that the lesions having SUVmax <2 were difficult to localize using SUVmax only. To our knowledge, this is the first report to show the use of SUVmax as an identifier of lesion on FDG PET-CT.

The pixel data was stored in DICOM files in a 16-bit integer form for all 3 scanners investigated. A 16-bit integer can express 65,536 different values. Since the number of voxels in the whole-body image may be around 10^7^, theoretically > 100 voxels on average may have exactly the same value. In fact, however, the distribution of SUV was quite skewed, as shown in Figure 8. It is reasonable that many voxels were detected when a smaller SUVmax was given, whereas only single voxel was detected when a larger SUV (e.g., >5) was given. In Figure 5, the number of voxels suddenly dropped once SUVmax became larger than 10. This can be explained as follows. In this study, we used DP instead of significant figures. They are slightly but clearly different. DP means the number of digits located to the right of the decimal point. Significant figures refers to the total number of digits irrespective of the decimal point location. For example, 9.8 is 1st DP and 2 significant figures, whereas 12.3 is 1st DP and 3 significant figures. Since 12.3 has more information than 9.8, fewer voxels were included within the range.

The effect of local maximum restriction was significant. The number of voxels that can become local maxima depends on the noise level of the image. Mathematically, when 3 × 3 × 3 restriction was applied, at most 1 of 2 voxels in each axis could become local maxima, indicating that 1/8 or a smaller number of voxels could become local maxima. Similarly, when 5 × 5 × 5 restriction was applied, at most 1 of 3 voxels in each axis and thus 1/27 or a smaller number of voxels could become local maxima. We did not try 7 × 7 × 7 restriction because we were worried that it might prevent identification of the voxel of SUVmax, considering that a single voxel size is 4 mm, and its diagonal is $4\sqrt{3}=6.9$ mm, and thus 7 voxels account for as large as 48.5 mm.

Some may argue that use of the 3rd or higher DP for SUVmax is redundant for daily radiological reports. That is true. SUV calculation uses body weight and the precision of body weight measurement may be 3 significant figures (e.g., 56.7 kg) or less. Radioactivity dosage measurements may introduce some errors. Furthermore, SUV varies depending on various technical (e.g., scanner, acquisition protocol, reconstruction protocol) and physiological (e.g., fasting conditions) factors. Therefore, the number excessive fine number is meaningless in diagnosis and treatment planning. Those may be why SUVmax is often written to the 1st DP (e.g., 3.1). However, in order to permit the future use of SUVmax as an identifier, we would like to propose that SUVmax be written as precisely as the PET-CT viewer allows. As mentioned before, this use of SUVmax would allow the diagnostic report to be summarized as a single image (Figure 1). In addition, it may also help radiologists to locate a lesion mentioned in a previous report so as to compare between past and present images. Our ultimate goal is to build a massive training dataset based on diagnostic reports and corresponding images. Writing the coordinate values (x, y, z) in the reports will be the best way to transfer the information to artificial intelligence. Currently, that may not be possible in most viewers and reporting systems. Also, the appearance of such information in the middle of a report may distract readers, and thus an automated system is needed to hide this information when humans read the report.

In the sub-analysis, we observed that the success rate was affected by some other factors. More specifically, the success rate was higher when the patient was younger or the injected dosage (MBq/kg) was larger. To reveal the reasons, we calculated Pearson's correlation coefficients between SUVmax vs. these factors, but there were no significant correlations. Thus, the underlying mechanisms remain unclear and will be investigated in future studies.

Although some radiological reports may be written with SUVmean, we did not try using SUVmean as a lesion identifier. Technically, SUVmean can be used instead of SUVmax for our current method; however, SUVmean calculation for every location is a time-consuming process and thus may not be practical. In addition, the range of SUVmean is smaller than SUVmax, which makes SUVmean less feasible for a lesion identifier.

The use of SUVmax is specific for PET. Although the maximum voxel value may not often be useful for CT or MRI, the idea could be applied to the apparent diffusion coefficient (ADC) images derived from diffusion weighted imaging of MRI, because the minimum of ADC is meaningful for diagnosis.

As limitations of the current study, we did not investigate the SUVmax shown in different image viewers. In some viewers, PET volumes are reconstructed (resliced) in the CT alignment, making slight changes to SUVmax. Secondly, we did not directly use the diagnostic reports but reviewed the images to re-measure SUVmax. Thus, we could not estimate the number of actual cases in which the SUVmax written in the reports could successfully locate the lesion. Such a study needs to be carried out. Thirdly, we investigated only 30 cases for this preliminary study. A larger study will be needed to confirm the results. In addition, head and neck cancer accounted for a large portion of the current study population, which does not necessarily reflect general population undergoing PET-CT. Finally, diagnostic reports often provide anatomical terms in the same sentence with SUVmax. This would be great information for selecting the appropriate location when SUVmax suggests several candidates, as in Figure 3. Such a method will be tested in future studies.

## Conclusion

The data suggested that SUVmax can be used as an identifier of lesion on FDG PET-CT. For this purpose, it is important that SUVmax is given precisely (3rd DP or more) and that local maximum restriction is applied to identify the voxel. The lesions showing SUVmax < 2 were difficult to identify. As this is a preliminary study investigating a small population from a single center, a larger study with many more patients will be needed to validate the results. The proposed method may have potential to make use of diagnostic reports retrospectively for constructing training datasets for AI.

## Data Availability Statement

The raw data supporting the conclusions of this article will be made available by the authors, without undue reservation.

## Ethics Statement

The studies involving human participants were reviewed and approved by Institutional Review Board of Hokkaido University Hospital for Clinical Research. Written informed consent from the participants' legal guardian/next of kin was not required to participate in this study in accordance with the national legislation and the institutional requirements. All procedures performed in this study involving human participants were in accordance with the ethical standards of the institutional review board (017-0454) and with the 1964 Helsinki Declaration and its later amendments or comparable ethical standards (EudraCT nr, 2017-003461-96).

## Author Contributions

KH and OM designed experiments, performed experiments, wrote and revised the manuscript. KM and SF collected image data. TS and KK wrote and revised the manuscript. All authors read and approved the final manuscript.

## Conflict of Interest

The authors declare that the research was conducted in the absence of any commercial or financial relationships that could be construed as a potential conflict of interest.

## Abbreviations

DP: decimal places
FDG: fluorodeoxyglucose
IQR: interquartile range
OSEM: ordered subsets expectation maximization
QIBA: Quantitative Imaging Biomarkers Alliance
SUV: standardized uptake value
VOI: volume of interest.

## References

[1] Ben-Haim S Ell P. 18F-FDG PET and PET/CT in the evaluation of cancer treatment response. J Nucl Med. (2009) 50:88–99. 10.2967/jnumed.108.054205 19139187

[2] Elsinga P Delaloye AB Chiti A Vandenberghe S Giammarile F Carrio I. Endorsement of international consensus radiochemistry nomenclature guidelines. Eur J Nucl Med Mol Imaging. (2019) 46:1399. 10.1007/s00259-018-4243-5 30989249

[3] Haberkorn U Strauss LG Dimitrakopoulou A Engenhart R Oberdorfer F Ostertag H. PET studies of fluorodeoxyglucose metabolism in patients with recurrent colorectal tumors receiving radiotherapy. J Nucl Med. (1991) 32:1485–90. 1714497

[4] Griffeth LK Dehdashti F McGuire AH McGuire DJ Perry DJ Moerlein SM. PET evaluation of soft-tissue masses with fluorine-18 fluoro-2-deoxy-D-glucose. Radiology. (1992) 182:185–94. 10.1148/radiology.182.1.1727280 1727280

[5] Adler LP Blair HF Makley JT Williams RP Joyce MJ Leisure G. Noninvasive grading of musculoskeletal tumors using PET. J Nucl Med. (1991) 32:1508–12. 1869970

[6] Wahl RL Jacene H Kasamon Y Lodge MA. From RECIST to PERCIST: evolving considerations for PET response criteria in solid tumors. J Nucl Med. (2009) 50 (Suppl. 1):122s–50s. 10.2967/jnumed.108.057307 19403881 PMC2755245

[7] Kim CK Gupta NC Chandramouli B Alavi A. Standardized uptake values of FDG: body surface area correction is preferable to body weight correction. J Nucl Med. (1994) 35:164–7. 8271040

[8] Nozawa A Rivandi AH Kesari S Hoh CK. Glucose corrected standardized uptake value (SUVgluc) in the evaluation of brain lesions with 18F-FDG PET. Eur J Nucl Med Mol Imaging. (2013) 40:997–1004. 10.1007/s00259-013-2396-9 23571761

[9] Kitao T Shiga T Hirata K Sekizawa M Takei T Yamashiro K. Volume-based parameters on FDG PET may predict the proliferative potential of soft-tissue sarcomas. Ann Nucl Med. (2019) 33:22–31. 10.1007/s12149-018-1298-0 30196378

[10] Kitao T Hirata K Shima K Hayashi T Sekizawa M Takei T. Reproducibility and uptake time dependency of volume-based parameters on FDG-PET for lung cancer. BMC Cancer. (2016) 16:576. 10.1186/s12885-016-2624-3 27484805 PMC4969656

[11] Krak NC Boellaard R Hoekstra OS Twisk JW Hoekstra CJ Lammertsma AA. Effects of ROI definition and reconstruction method on quantitative outcome and applicability in a response monitoring trial. Eur J Nucl Med Mol Imaging. (2005) 32:294–301. 10.1007/s00259-005-1926-5 15791438

[12] Nishiyama Y Kinuya S Kato T Kayano D Sato S Tashiro M. Nuclear medicine practice in Japan: a report of the eighth nationwide survey in 2017. Ann Nucl Med. (2019) 33:725–32. 10.1007/s12149-019-01382-5 31236776

[13] Niederkohr RD Greenspan BS Prior JO Schoder H Seltzer MA Zukotynski KA. Reporting guidance for oncologic 18F-FDG PET/CT imaging. J Nucl Med. (2013) 54:756–61. 10.2967/jnumed.112.112177 23575994

[14] Dalal S Hombal V Weng WH Mankovich G Mabotuwana T Hall CS. Determining follow-up imaging study using radiology reports. J Digit Imaging. (2019) 33:121–30. 10.1007/s10278-019-00260-w 31452006 PMC7064667

[15] Liu Y Liu Q Han C Zhang X Wang X. The implementation of natural language processing to extract index lesions from breast magnetic resonance imaging reports. BMC Med Inform Decision Making. (2019) 19:288. 10.1186/s12911-019-0997-3 31888615 PMC6937920

[16] Spandorfer A Branch C Sharma P Sahbaee P Schoepf UJ Ravenel JG. Deep learning to convert unstructured CT pulmonary angiography reports into structured reports. Eur Radiol Exp. (2019) 3:37. 10.1186/s41747-019-0118-1 31549323 PMC6757071

[17] Piotrkowicz A Johnson O Hall G. Finding relevant free-text radiology reports at scale with IBM Watson content analytics: a feasibility study in the UK NHS. J Biomed Semantics. (2019) 10:21. 10.1186/s13326-019-0213-5 31711538 PMC6849164

[18] Banerjee I Bozkurt S Caswell-Jin JL Kurian AW Rubin DL. Natural language processing approaches to detect the timeline of metastatic recurrence of breast cancer. JCO Clin Cancer Inform. (2019) 3:1–12. 10.1200/CCI.19.00034 31584836

[19] Bogaerts J Ford R Sargent D Schwartz LH Rubinstein L Lacombe D. Individual patient data analysis to assess modifications to the RECIST criteria. Eur J Cancer. (2009) 45:248–60. 10.1016/j.ejca.2008.10.027 19095437

[20] Nguyen NC Vercher-Conejero JL Sattar A Miller MA Maniawski PJ Jordan DW. Image quality and diagnostic performance of a digital PET prototype in patients with oncologic diseases: initial experience and comparison with analog PET. J Nucl Med. (2015) 56:1378–85. 10.2967/jnumed.114.148338 26159588

[21] Hirata K Kobayashi K Wong KP Manabe O Surmak A Tamaki N. A semi-automated technique determining the liver standardized uptake value reference for tumor delineation in FDG PET-CT. PLoS ONE. (2014) 9:e105682. 10.1371/journal.pone.0105682 25162396 PMC4146536

[22] FDG-PET/CT Technical Committee. FDG-PET/CT as an Imaging Biomarker Measuring Response to Cancer Therapy, Quantitative Imaging Biomarkers Alliance, Version 1.13. Technically Confirmed Version. Available online at: http://qibawiki.rsna.org/images/1/1f/QIBA_FDG-PET_Profile_v113.pdf. RSNAORG/QIBA (accessed November 18, 2016).

