# Supplementary material for: A Preliminary Study to Use SUVmax of FDG PET-CT as an Identifier of Lesion for Artificial Intelligence
Source: Front Med (Lausanne). 2021 Apr 28;8:647562. doi: 10.3389/fmed.2021.647562 (PMC8113693; doi:10.3389/fmed.2021.647562)
Supplement: Supplementary file 1 [file Presentation_1.PPTX]

## Slide 1
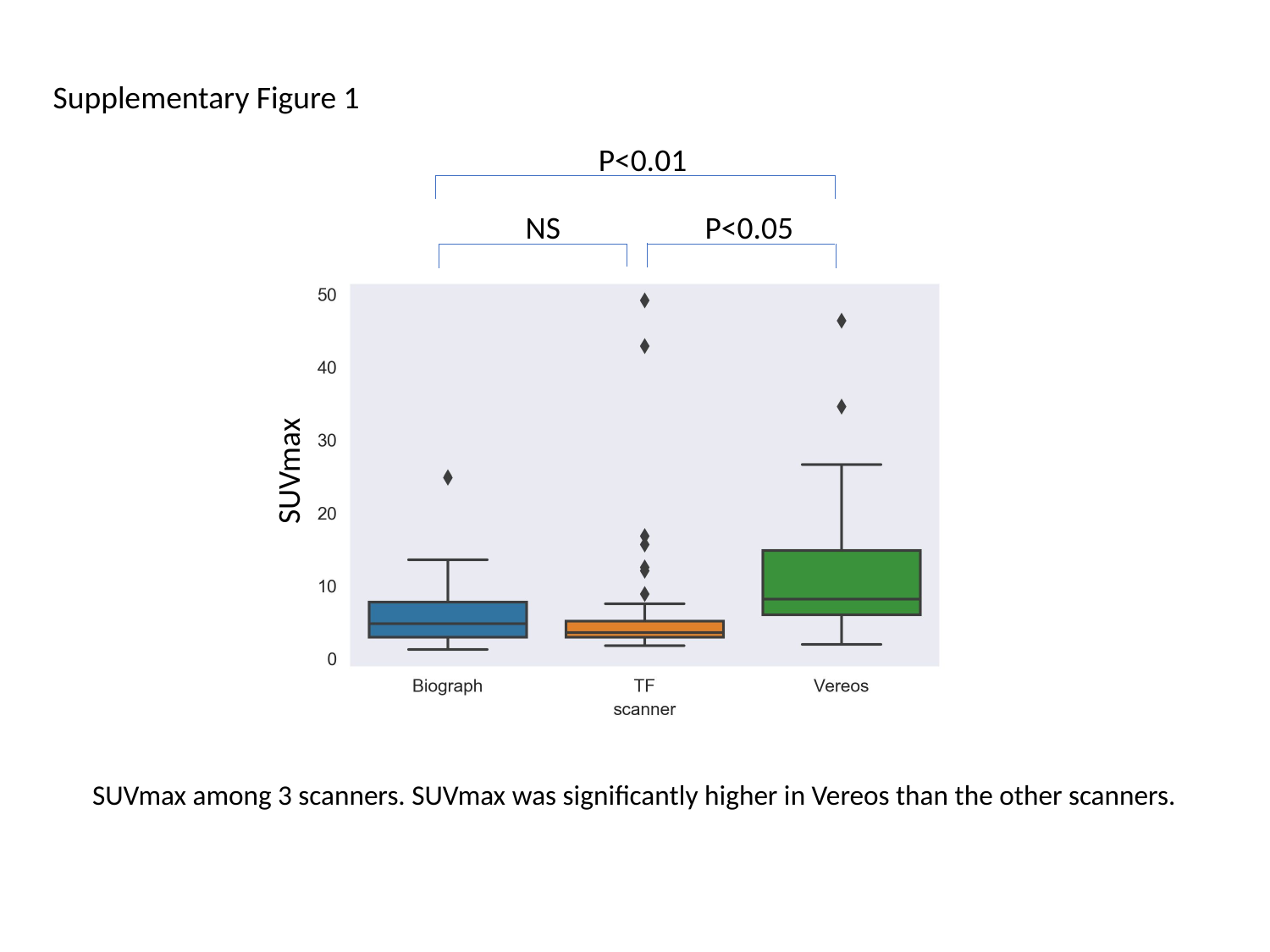

Supplementary Figure 1
P<0.01
NS
P<0.05
SUVmax
SUVmax among 3 scanners. SUVmax was significantly higher in Vereos than the other scanners.
